# Patent foramen ovale as a rare cause of sudden refractory hypoxemia: a case report

**DOI:** 10.3389/fcvm.2025.1715242

**Published:** 2025-12-08

**Authors:** Haytham Allaham, Samuel Bennett, Mark Sonbol, Rahul Annabathula, Brian Barr

**Affiliations:** Division of Cardiovascular Medicine, University of Maryland Medical Center, Baltimore, MD, United States

**Keywords:** patent foramen ovale, refractory hypoxemia, intracardiac shunt, intracardiac echocardiography, orthodeoxia

## Abstract

An 80-year-old man presented with progressive dyspnea and severe hypoxemia refractory to high-flow oxygen therapy and was found to have a large persistent patent foramen ovale (PFO)-mediated right-to-left shunt (RTLS). The patient underwent successful percutaneous PFO closure via a 35 mm Amplatzer septal occluder guided by intracardiac echocardiography (ICE), resulting in complete resolution of his hypoxia within 24 h. This case report highlights the workup of systemic refractory hypoxemia, the importance of cardiac shunt physiology in considering an RTLS, and the utility of PFO closure under ICE guidance. For patients with a PFO-mediated RTLS, percutaneous closure is an effective therapeutic option.

## Introduction

In the developing fetus, the foramen ovale serves as a one-way valve that allows blood to pass from the right atrium (RA) to the left atrium (LA), thereby bypassing the high-resistance circuit of the fluid-filled lungs. After birth, the foramen ovale typically closes functionally when left atrial pressure exceeds right atrial pressure; however, anatomical (structural) fusion of the septum primum and septum secundum may be delayed for weeks to up to a year, and incomplete fusion resulting in a persistent patent foramen ovale (PFO) has been reported in 20%–25% of adults (1). While most people with PFO never develop symptoms, its presence increases the risk for a paradoxical embolism, whereby a clot travels from the venous to arterial circulation (2). Moreover, in rare cases with elevated right atrial pressures, hypoxemia can develop if sufficient deoxygenated blood travels from the RA to the LA, a phenomenon known as a right-to-left shunt (RTLS) (3). Intracardiac RTLS-mediated hypoxemia is a rare complication of PFO for which percutaneous closure is a safe and effective treatment (4). We describe herein the case of an elderly man who presented with severe, refractory hypoxemia attributable to a large, sudden PFO and who underwent PFO closure with full resolution of his hypoxemia within 24 h of the procedure.

## Case presentation

An 80-year-old man with progressive dyspnea on exertion and hypoxia (SpO_2_ 78%–82%) presented to the emergency department after being referred by his primary care physician (PCP), who detected the hypoxia during an annual screening. The patient had no prior history of hypoxia and was doing his usual activities the day before. His medical history includes obstructive sleep apnea (OSA), depression, mild chronic obstructive pulmonary disease, and a 45-pack-year smoking history with 20 years of abstinence. A transthoracic echocardiogram (TTE) conducted 8 months prior demonstrated a normal ejection fraction, normal right ventricular (RV) size and function, normal RA, and evidence of right-to-left shunts on color Doppler.

Two weeks prior to presentation, he had suffered a fall while walking his dog. Since then, he had been experiencing progressive dyspnea on exertion and left-sided chest pain with deep inspiration. He denied any fever, chills, productive cough, or swelling of the lower extremity. Apart from resting hypoxia to 85% on room air, vitals, physical exam, and labs were unremarkable. Of note, the patient's oxygen saturation was normal during a prior PCP clinic visit earlier that year. An electrocardiogram demonstrated normal sinus rhythm with left axis deviation, left ventricular (LV) hypertrophy, and left anterior fascicular block. Computed tomography angiography (CTA) of the chest showed no pulmonary emboli, pneumothorax, or evidence of significant intraparenchymal lung disease, but multiple rib fractures and a small, loculated effusion in the left chest. After admission, he had increasing oxygen requirements, necessitating 100% high-flow oxygen to maintain SpO_2_ saturations in the high 80% range. A recent pulmonary function test demonstrated the presence of mild COPD (FEV1/FVC 78%) with no significant restrictive lung disease (total lung capacity, 5.4 L; diffusing capacity for carbon monoxide, 76%). A limited TTE with agitated saline was suggestive of a right-to-left atrial-level shunt, normal LV systolic function, and normal RV size and function. On hospital day 4, he underwent right heart catheterization, which showed minimally elevated right-sided filling pressures (RA 10 mmHg, RV 32/6 mmHg) and mild pulmonary hypertension [pulmonary artery (PA) 32/19, mean 25 mmHg] with no oxygen “step-up” on cardiac shunt run [inferior vena cava (IVC) 39%, superior vena cava (SVC) 36%, RA 35%, RV 36%, PA 36%], ruling out significant left-to-right shunt. The patient's hypoxia was initially attributed to hypoventilation secondary to pain from the rib fractures. However, despite pain management and reported improvement in breathing, his oxygen requirements remained significant. On hospital day 5, he was transferred to our institution for further evaluation of the intracardiac shunt as the primary etiology of his persistent, worsening acute hypoxic respiratory failure.

At our institution, a repeat TTE with agitated saline demonstrated a right-to-left shunt that worsened on sitting upright. Clinical orthodeoxia was noted, consistent with an intracardiac shunt; however, formal position-dependent oxygen saturation measurements were not recorded. Repeat CTA demonstrated no evidence of arteriovenous malformations.

**Figure 1:**
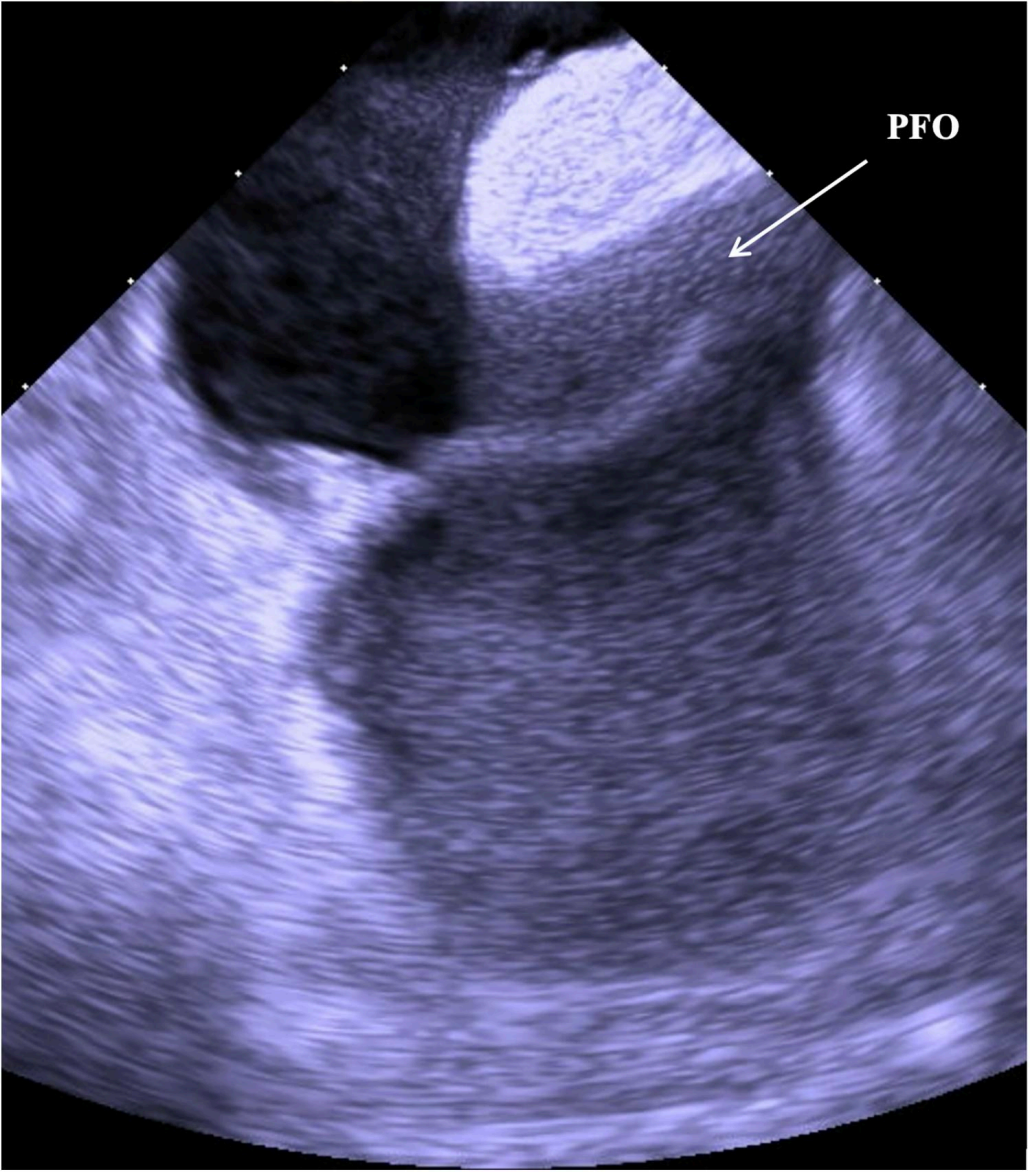
ICE image demonstrating a large PFO with a wide and elongated tunnel.

**Figure 2:**
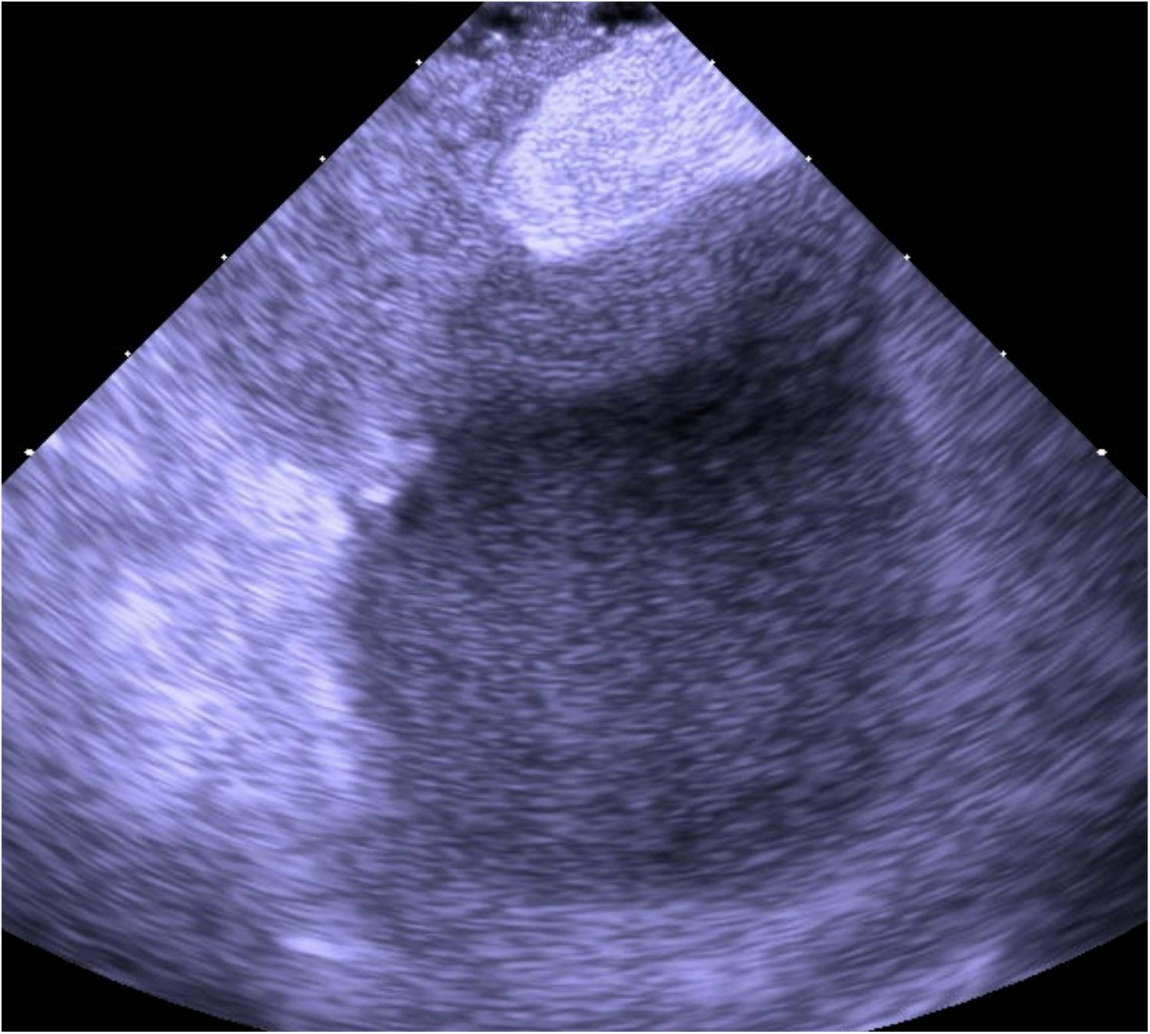
ICE with agitated saline contrast showing immediate right-to-left passage of microbubbles through the PFO, confirming a significant interatrial shunt.

**Figure 3:**
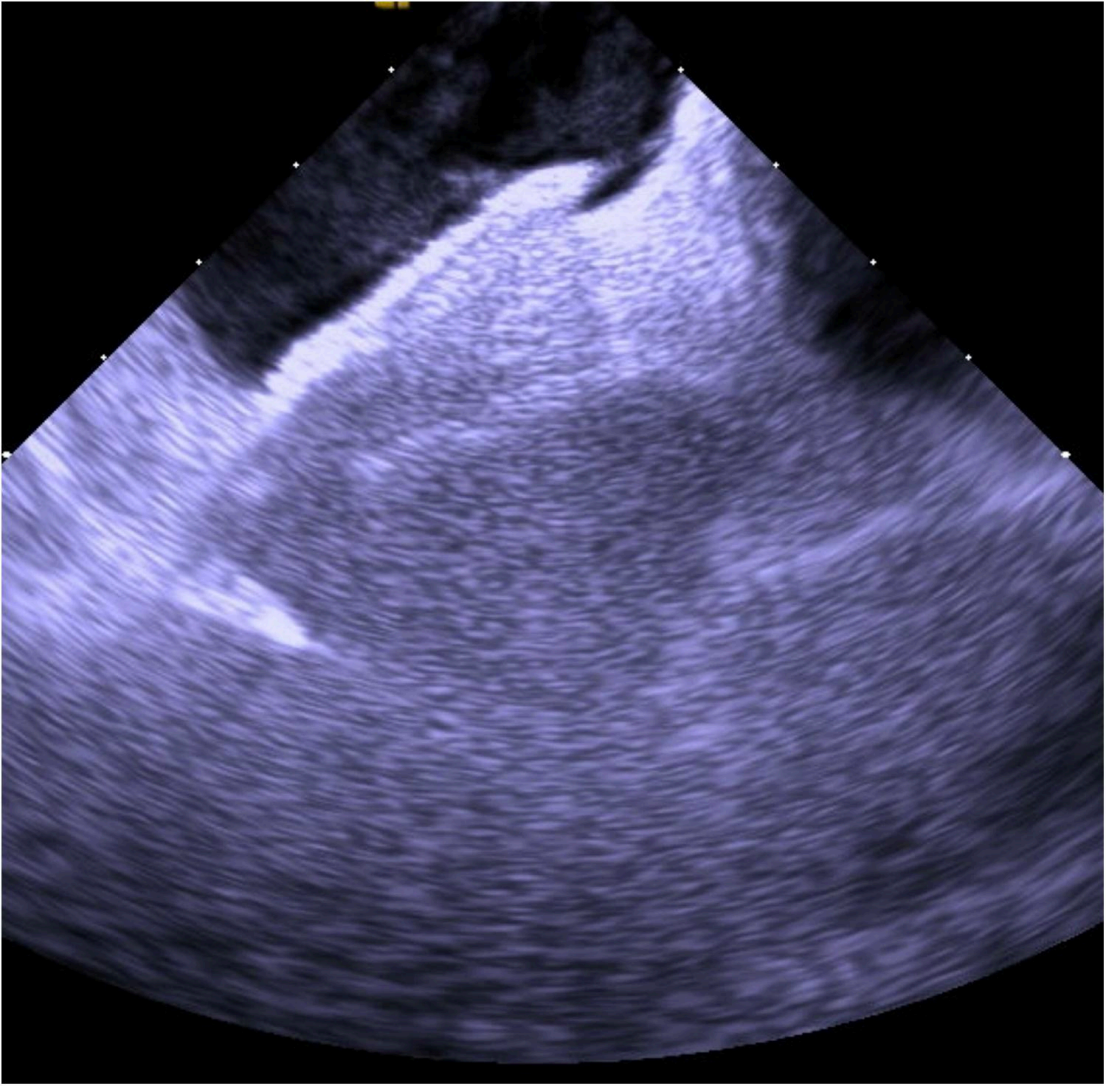
Post-closure ICE demonstrating the Amplatzer septal occluder device in optimal position with complete elimination of interatrial shunting.

Based on the available evidence, a multidisciplinary team had concluded that a PFO-mediated RTLS was the primary driver of the hypoxia. The patient was referred for intracardiac echocardiographic (ICE) evaluation for possible PFO closure. ICE evaluation demonstrated a large PFO with extensive right-to-left shunting on intravenous bubble study (Supplementary Videos S1, S2; Figures 1, 2). The PFO measured >5 mm in width and >10 mm in length, with no evidence of significant septal aneurysmal changes. Pulmonary vein oxygen saturation was recorded at 97% and LA saturation at 83%. Successful closure of the PFO was achieved using a 35 mm Amplatzer septal occluder device. No residual shunt was observed on agitated saline contrast testing after occluder placement (Supplementary Video S3; Figure 3). The patient was successfully weaned off supplemental oxygen within 24 h and exhibited a normal oxygen saturation of 98% on room air the next morning. He was discharged home in a stable and improved condition that day. A TTE performed 6 months after discharge demonstrated no residual shunt.

## Discussion

Refractory hypoxemia is a condition characterized by persistent low levels of oxygen in the blood despite supplemental oxygen administration and adequate ventilation. The mechanisms of refractory hypoxemia are complex and involve various factors impairing oxygenation, which include ventilation–perfusion mismatch, hypoventilation, impaired diffusion, intracardiac shunts, and low inspired oxygen partial pressure (5).

This case report not only illustrates the diagnostic workup of refractory hypoxemia but also adds to the growing body of literature featuring patients with underlying lung disease and intracardiac shunts who have hypoxemia disproportionate to their lung disease (1). In this case, arterial blood gas ruled out hypoventilation, and our patient was receiving 100% high-flow oxygen, ruling out low inspired partial pressure as a cause. Extensive diagnostic workup, including V/Q perfusion scan, chest CTA, and a right heart catheterization, ruled out V/Q mismatch, diffusion impairment, and pulmonary shunt physiology.

While our patient perplexedly had no history of congenital cardiac defects and had a normal TTE earlier that year, the presence of a sudden PFO on repeat echocardiogram and the low severity of his comorbid hypoxemia-related conditions (OSA, COPD) supported RTLS as the only plausible explanation for the refractory hypoxemia. The septal anatomy noted on ICE was suggestive of a large PFO rather than an atrial septal defect. The PFO tunnel was noted as wide (>5 mm) and long (>10 mm) on ICE imaging. The diagnosis of a ruptured PFO is further supported by the complete resolution of the patient's hypoxemia within 24 h of the PFO closure with a 35 mm Amplatzer occluder device.

The temporal association between the chest trauma and the onset of hypoxemia in our patient can be explained by a functional rather than an anatomical change. Pain-limited ventilation and rib-related chest wall mechanics can increase intrathoracic pressure and promote intermittent atelectasis, both of which may lead to modest elevations in right-sided pressures. In the presence of a PFO whose anatomy favors streaming of venous return toward the interatrial septum, even small and transient right-to-left pressure differences can permit clinically significant shunting. Thus, the trauma likely acted as a trigger that unmasked an otherwise silent PFO-mediated right-to-left shunt, rather than being the primary cause of the defect itself.

Only a few studies have been published on the relationship between PFO and severe arterial deoxygenation syndromes (4, 6–14). Any condition that can elevate right-sided heart pressure, such as OSA, COPD, and pulmonary hypertension, can increase RTLS (15). Indeed, case reports have demonstrated a relationship between certain hypoxemia-associated conditions and PFO, where patients developed hypoxemia disproportionate to the severity of their primary pulmonary disease (1, 3). Moreover, several anatomical variants (e.g., persistent prominent Eustachian valve) can cause significant shunt, even in the presence of a normal mean RA pressure (15).

The invasive standard for PFO diagnosis is transesophageal echocardiography with agitated saline contrast, although transthoracic echocardiography (TTE) with agitated saline contrast, while less accurate, may also be used (3, 16).

Based on the results of multiple clinical trials, PFO closure is currently recommended for the secondary prevention of paradoxical embolic stroke in certain patient populations (2, 16). However, the role of PFO closure in treating hypoxemia is not well established. To date, no randomized clinical trials have been performed that address the role of percutaneous PFO closure in desaturation syndromes. Pristipino et al. (15) published a recent expert position paper on PFO closure that was prepared with the involvement of eight European scientific societies. They purported the benefits that percutaneous PFO closure can have in “select patients with arterial hypoxemia syndrome.” They recommended, when appropriate, proposing PFO closure with shared decision-making while being sure to underscore the lack of evidence. While the procedure has been consistently demonstrated to be efficacious and safe, with low complication rates, procedural success is contingent on confirming that the RTLS is the primary cause of the hypoxemia (4).

## Conclusion

A small subset of patients with PFO, whether due to elevated right-sided filling pressures or acquired anatomical abnormalities, can develop systemic hypoxemia. Diagnostic workup of refractory hypoxemia in patients with imaging negative for intraparenchymal diseases causing V/Q mismatch or diffusion impairment should also include TTE with bubble study as a screening test for shunt physiology. If the pretest probability is high that the hypoxemia is primarily driven by an intracardiac RTLS, such as a PFO, ICE-guided percutaneous closure can be performed as a safe and effective means of treating the hypoxemia.

## Data Availability

The original contributions presented in the study are included in the article/Supplementary Material, further inquiries can be directed to the corresponding author/s.
